# Life Design for Youth as a Creativity-Based Intervention for Transforming a Challenging World

**DOI:** 10.3389/fpsyg.2021.662072

**Published:** 2021-05-04

**Authors:** Luca Fusco, Anna Parola, Luigia Simona Sica

**Affiliations:** Department of Humanities, University of Naples Federico II, Naples, Italy

**Keywords:** career support, life design, career adaptability, creativity, transformability, stagnating economies

Living the present decade as a young person means facing challenges never experienced by any other young generation in history. Changes from the last 2 years in the global scenario (mainly linked to the Covid-19 pandemic) have brought new major issues to a world which already presented several threatens to the future. The consequences of the pandemic are likely to harm even more the future of young people, already seriously undermined by the risks related to climate changes, social inequalities, and work issues (global youth unemployment rates never recovered from 2008, Masenya, 2021). This may be particularly true in countries with stagnating economies like Italy, where the effects of the last economic crisis and changes related to the flexibilization of the work market have been dramatic for the careers and lives of young people (Parola and Donsì, 2018; Liotti, 2020).

In this critical situation, educational systems need to help young people to think about their future and find their way to build prosperous and sustainable lives (De Vos et al., 2020).

Career support is the term that indicates any kind of practice (career guidance, career education, career counseling) aimed to help the individual to cope with career decisions and career design processes (Bimrose and Brown, 2020). Career practitioners structure their interventions basing on the needs of the contexts where they operate, their material resources and their theoretical view of the career development phenomenon. Traditionally, in the last century, career practitioners' activities followed what has been called the “person-environment fit approach,” that is, they were aimed to assess people's skills, interests, and other characteristics to diagnose, and subsequently prescribe the job environment where they could better perform (Su et al., 2015). After dominating the career field for almost half a century, the popularity of the person-environment fit approach has decreased, leaving the space to a new leading paradigm which emerged at the end of the 20th century: the Life Design paradigm (Savickas et al., 2009; Savickas, 2012). Life Design conceives career as a self-directed process, through which the individual, negotiating personal meaning in social interactions, shapes her/his own life course.

Contesting the neo-positivist perspective of old career theories, the new approach embraces a constructivist view of the life course and encourages the individuals to actively engage in the identity construction process and develop general skills that can lead them to successfully interact with the environment.

Life Design appears to be more useful for career support than the “person-environment fit” approach to career guidance for several reasons.

First, all over the world, the labor market is far more unpredictable than how it was decades ago. The economic context is increasingly failing either to maintain or create safe spaces for the young people (Guichard, 2013; Savickas, 2015; Hartung, 2016; Maree, 2020). Career paths are no longer predictable, secure (Herr, 2008) and describable by linear trajectories (Larson, 2011). Contemporary workers, during their life course, go through an indefinite series of transitions. Focusing on the development of transitions and planning related skills is far more appropriate than prescribing career choices. Career identity has become the dynamic outcome of a continuous negotiation between the self and the context. In this sense, focusing on improving career skills Life Design aims to have a long-term effect on the individuals' life course.

Second, from an ethical point of view Life Design promotes the freedom of the individual to choose her/his own career and decide her/his positioning in the world. Workers are no longer encouraged to passively receive a career identity. They are supported to build actively and freely a professional self which can contribute to their personal satisfaction and which can match their vision of the world, integrating with a pre-existing sense of identity. This sense of identity allows the individuals to develop their planfulness (Robitschek et al., 2012) in line with what is relevant to them as human beings. That is, their right to authenticity is promoted (Vieira et al., 2018). Moreover, they are stimulated to actively reflect on the relationship between the individual and the social environment, questioning the unconscious self-imposed limitations related to the influence of a role ascribed by the social structure (Guichard, 2004; McIlveen and Patton, 2007; Fusco et al., 2020a).

In order to clarify which kind of skills are to be improved for career success according to the Life Design approach, the construct of career adaptability was developed (Savickas, 1997; Savickas and Porfeli, 2012). Career adaptability, in this perspective, is intended as a fundamental prerequisite for managing career paths. It can be described as the ability to easily change in line with what is required by the environment in the different moments of the career paths. Five psychological skills, “the 5 Cs” (namely control, confidence, concern, curiosity, and commitment) have been identified by career scholars as major components of career adaptability (Savickas et al., 2009; Savickas and Porfeli, 2012). Studies have shown that career adaptability is a precious resource for development, predicting positive outcomes as life satisfaction (Santilli et al., 2017; Parola and Marcionetti, 2021) and future orientation (Cabras and Mondo, 2018).

In addition to this, studies have also identified a large set of resources as somehow composing adaptability or related to the career adaptation process (Nota et al., 2014). Planning abilities, optimism, resilience, curiosity, and many others have been identified as positive elements for career development in this perspective. Among this set of useful dimensions, creativity can be considered an important factor to be highlighted for the life design paradigm.

Creativity is a fundamental resource for constructing an identity when the environment seems to offer limited chances for the future. According to the classical conception of Williams (1980, 1994) it includes different elements: (1) the tendency to look at problematic situations with unexpected solutions (complexity), (2) the tendency to visualize mental images of possible situations (imagination), (3) the tendency to act under unstructured conditions for defending personal ideas (risk-taking).

The purpose of using creative resources in career interventions is not new (McMahon, 2016). Creativity has been imagined as a potentially helpful element for the exploration of possible future selves and the definition of a personal career identity (SIOT model; Aleni Sestito and Sica, 2016) and for decision-making processes (de Valverde et al., 2017). Life Design conceives career transitions as the product of self-constructed personal narratives. Creativity, allowing the individual to author his life story in an original way, can help the individual to design his future and flexibly overcome the difficulties coming from her/his environment (de Valverde et al., 2017).

We recognize the importance of referring to the Life Design paradigm in preparing career interventions. Nevertheless, in this paper, we intend to highlight problematic issues coming from the application of this approach to the interventions for young people facing the contemporary world challenges and to propose possible elements which could integrate Life Design interventions.

Despite putting the individual's relationship with the environment as a fundamental component of career success, many scholars were critical with the Life Design approach for proposing a universal idea of career intervention for the globalized world in the XXI century, which may not be in line with the specific characteristics of the contexts where it is implemented (Ribeiro and Fonçatti, 2018). In this sense, promoting a flexible approach to career construction for young people in environments with high risk for their material future may be, by itself, dangerous for their mental health (Fusco et al., 2020b; Parola and Marcionetti, 2020) and for the construction of their career identity (Fusco et al., 2019).

Skepticism about positive changes in society, cynicism and even hopelessness may be the harmful psychological outcomes that young people might develop in such environments. These elements could represent an obstacle for the exploration and construction of their vocational identity, and the development of a plan of action for achieving their life goals. Individual processes enhanced by Life Design interventions might be undermined by the awareness and preoccupations that young people have about the difficulties related to the achievement of career goals. For this reason, we believe that, in career interventions proposed for young people living the current historical time, goals related only to career adaptability may be inadequate for their needs.

We think that career support needs to consider the problems related to the unpleasant perspective for their future that young generations may experience in the current historical time. Life Design proposes to act on the subject/environment relationship helping individuals to develop the abilities needed to fit the contemporary job market. This view of career development may bring young people to ask: “should we to fit a world which seems to fall apart?.”

During adolescence, people develop the skill to think about how things could be better in the environment they live in. Specifically, the achievement of formal thinking allows the adolescent to think of reality as only one of the possibilities of what could be. During this life period, as noted by Piaget (1965), individuals have frequently the tendency to go through a “messianic stage.” That is, a time in life when adolescents unrealistically think they know how the world should change and be reformed to solve its problems.

A possible solution for support young people to face a hostile job market is the inclusion, in career guidance actions, of activities that stimulate creative thinking related to the desire for the creation of the environment young people would like to live in.

We think that creative skills are not only useful for the individuation of a personal developmental path. Divergent thinking, complexity, risk-taking, and curiosity are skills which can also help to imagine alternative worlds, supporting the design of modification of external conditions. In this perspective creativity would serve the individual's ability to understand and modify both the self and the environment where she/he lives in.

The creative exercises which should be proposed by career interventions would help young people to play with their ability to picture and desire world scenarios. Moreover, these interventions would aim to support the students to include their possible careers as engines for the realization of the desired environmental changes. Activities should stimulate students to answer questions like:

1. In which kind of city/country/world would you like to work in? Which are the characteristics of the environment in which you would like to live?
2. How would your career choices help you to live in such an environment?
3. How can your career choices help the environment in which you live now to develop your desired characteristics?

Such activities would help young people's creativity to design life paths that conciliate the individual's personal plans with the possibility of a transformation of the environment. Young people would reflect about their desired position in society, their attitude toward future changes, their personal values and their social needs.

In these activities, workers must be described to young people not just as passive adaptors to environments with given characteristics, but as active choosers, creators, and transformers of environments. Their careers should be described as the way through which individual can build their desired relationship with the world. The enhancement of hope and a positive attitude toward the possibility of change would also be essential points of these activities.

Reflections produced by these types of career support would help the individual become more aware of her/his desired positioning toward possible actions that would affect her/his relationship with the environment:

1. The possibility of engaging in professional or personal activities that transform communities/cities/countries into better environments according to the personal view of the individual.
2. The possibility of moving to environments with different characteristics (migration).
3. The possibility of coping with challenges related to the difficulty of realizing desires.

Interventions following this suggestion would also stimulate the “5 Cs” for career adaptability: “curiosity” about one's environment and the possible way a career could make it better; “control” over the future of personal life and of the relationship one will have with the environment; “confidence” in the chance of influencing the environment through personal actions; “concern” about careers as tools for creating desirable future, “commitment” for life goals; related to personal values; interest and desires about the relationship with the world. In our perspective, creativity would represent the sixth “C” - resource for career, a central psychological precondition for being able to choose and design work and life environments.

Future studies should assess how this integration to life design inspired interventions would produce a positive change in career-related skills and other career and psychological outcomes. Moreover, these interventions may contribute to reach collective goals, stimulating individuals to design their careers for reaching positive achievements for the communities. They would improve the transformability of the social systems (Folke et al., 2010), the possibility of going toward a change of system in case of impossibility to realize the people's needs.

Nonetheless, these interventions would also support the individual to decide her/his own type of goals. Individuals would personally decide if they, reflecting on future world transformation, will just pursue the transformation of their close personal reality or if their career will contribute to a societal or even a global change.

## Author Contributions

LF, AP, and LS wrote the manuscript, critically revised it, and approved the final version. All authors contributed to the article and approved the submitted version.

## Conflict of Interest

The authors declare that the research was conducted in the absence of any commercial or financial relationships that could be construed as a potential conflict of interest.
